# Tight Binding of Transition-State Analogues to a Peptidyl-Aminoacyl-l/d-Isomerase from Frog Skin

**DOI:** 10.1002/cbic.201100203

**Published:** 2011-07-07

**Authors:** Verena Gehmayr, Christa Mollay, Lorenz Reith, Norbert Müller, Alexander Jilek

**Affiliations:** [a] Chair of Bioinformatics, Department of Biotechnology, University of Natural Resources and Life SciencesMuthgasse 18, 1190 Vienna (Austria); 2 Institute of Organic Chemistry, Johannes Kepler University LinzAltenberger Strasse 69, 4040 Linz (Austria); 3 Institute of Molecular Biology, Austrian Academy of SciencesBillrothstrasse 11, 5020 Salzburg (Austria)

**Keywords:** chirality, d-amino acids, enzyme catalysis, inhibitors, post-translational modifications

d-Amino acids in peptide linkage are a noteworthy exception from the universal homochirality of proteins and peptides.[1] The first animal peptide that was found to contain a d-amino acid as the second residue was dermorphin, isolated from the skin of a South American tree frog.[2] This peptide binds with high affinity to μ-opiate receptors. The d-residue in this and related amphibian opioid peptides is essential for the biological function, whereas the all-L-isomers lack activity.

Further studies led to the discovery of additional diastereomeric peptides from numerous sources,[3] which include two components, a natriuretic peptide and a β-defensin, from the venom of the male platypus, a primitive mammal.[4] All of these peptides contain a single d-amino acid substitution in a well-defined position, which is the second residue of the mature product in all vertebrate peptides known to date. Whenever the structure of the precursor polypeptides was deduced from cloned cDNAs, a codon for the corresponding L-amino acid was present in the mRNA at the position where a d-residue occurs in the mature form. This fact shows that the d-residue is enzymatically introduced during a post-translational event.[5]

Fire-bellied toads (*Bombina variegata*, *Bombina bombina* as well as *Bombina orientalis*) contain in their skin secretions bombinins H, peptides with antibacterial and hemolytic properties,[6] some of which contain a d-amino acid substitution; indeed, both isomers coexist in the skin glands. The d-amino acid at the second position gives rise to subtle changes in biophysical properties and folding propensity.[7] More recently, the d-amino acid was observed to affect both the target microbial cell selectivity and membrane-perturbing activity of bombinin H.[8] The peptidyl-aminoacyl-L*/*d-isomerase (henceforth briefly referred to as isomerase), which catalyses this post-translational stereo-inversion, has been purified and characterized.[9] Surprisingly, this protein, a 52 kDa glycoprotein, is apparently excised from a polyprotein containing several isomerase domains separated by short individual spacer sequences. Genes coding for related proteins are present in several vertebrate species.

In particular, the amino-terminal domain H of the human IgG-Fcγ binding protein[10] is related to the frog skin enzyme, and thus, could have isomerase activity. The similarity is highest in a central region, which contains two conserved histidine residues as well as a pair of cysteines. Further studies have shown that the *Bombina* isomerase is inactivated by diethylpyrocarbonate, which indicates that one or two histidine residues indeed are part of the active site. The pH dependence of the reaction rate, which shows a steep decline below pH 5.5, supports this notion. If the isomerization reaction, which proceeds in both directions, is carried out in tritiated water, radioactivity is incorporated into the second residue, which is an Ile in bombinin H. This demonstrates that the change of chirality proceeds through a deprotonation/protonation mechanism at the α-carbon. Such a mechanism is also operative in other isomerases, for example, an epimerase from spider venom,[11] which is structurally unrelated to the *Bombina* isomerase, and an isomerase from the venom of male platypus.[12] The latter could also involve at least one His as a catalytic base;[13] its sequence, however, is not known at present. The enzymes from hylid frogs, conus snails, and crustaceans as well as the isomerase activity, which is likely present in mouse heart, are not yet studied to sufficient detail.[14]

Here, we have extended these studies in order to obtain a closer insight into the reaction mechanism. For this purpose, we performed the reaction catalysed by the frog isomerase in ^2^H_2_O. A comparison of the reaction rate with the rates of deuterium incorporation revealed that the sole product is deuterated during early reaction stages (Figure S1 in the Supporting Information). These observations are consistent with a classical two-hydrogen-acceptor (two-base) mechanism (Scheme 1).[15] In D_2_O, the reaction was significantly retarded (by about three times when compared to water). This effect suggests that proton delivery to the α^2^-carbon also significantly contributes to the reaction rate. Moreover, the symmetry of the reaction is shifted at the cost of the L→d reaction as indicated by the equilibrium concentrations. This fact indicates that the action of the bases is differentially impaired by the isotope effect.

**Scheme 1:**
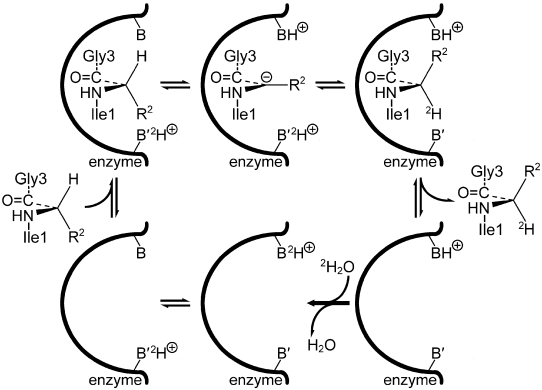
Proposed two-base-mechanism for the isomerase reaction. The two catalytic bases are represented by B and B′. When the reaction is performed in D_2_O, in the presence of substrate, enzyme-bound protons apparently do not exchange with solvent protons.

Next, we synthesized two specifically designed planar substrate analogues and tested their effect on the isomerization reaction catalysed by the frog isomerase. Peptides Ile-Δ^2, 3^-Ala-Gly-Pro-Val-Leu-amide (IΔA) and Ile-Δ^2, 3^-Phe-Gly-Pro-Val-Leu-amide (IΔF) both contain at the second position an α,β-unsaturated amino acid with a planar sp^2^ hybridised α-carbon, which eventually could mimic potential enolate anion intermediates (Scheme 2) or the planar transition states in a S_N_2-type mechanism (concerted deprotonation/protonation). The corresponding saturated peptides, IA and IF, are good substrates of the isomerase, yet with vastly diverse catalytic efficiencies ((*k*_cat_/*K*_M_)_IF_/(*k*_cat_/*K*_M_)_IA_≈50). Compounds IΔA and IΔF exerted a strong inhibitory effect independently of the direction of the enzymatic reaction (Figure 1), that is, either L→d or vice versa (Figure S2 in the Supporting Information).

**Scheme 2:**
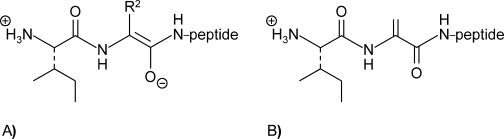
Structural comparison of: A) a hypothetical anionic isomerization intermediate, and B) inhibitor peptide IΔA.

**Figure 1:**
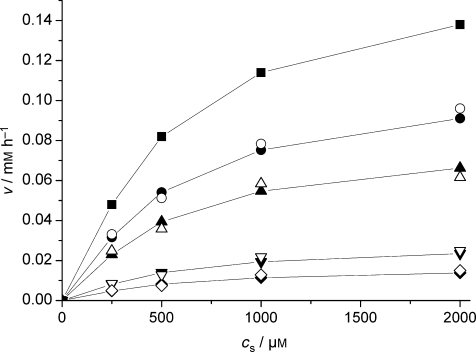
Initial reaction velocities as a function of substrate concentration at various inhibitor concentrations. Without inhibitor (▪) and in the presence of 0.5 μm (•), 1 μm (▴), 5 μm (▾) and 10 μm (◆) IΔF, or 0.5 μm (○), 1 μm (▵), 5 μm (▿) and 10 μm (◊) IΔA. Substrate was labelled peptide IF. See the Experimental Section for details.

Surprisingly, the experimental data indicated that the inhibition was not competitive; high substrate concentrations did not restore enzymatic activity as it is the characteristics of competitive inhibition (a Michaelis–Menten analysis of the data in Figure 1 is shown in Figure S3 in the Supporting Information). Furthermore, no evidence of reactivation of partly inhibited enzyme was observed after 100-fold dilution into buffer. Conversely, their action was virtually irreversible, and therefore, probably indicative of tight, yet noncovalent binding of the inhibitors.[16] The determination of the release rates (off-rate) of the inhibitors from the enzyme was, however, hampered by the rapid loss of the enzymatic activity in a diluted solution. Therefore, we cannot fully exclude the possibility that the Δ^2, 3^-peptides could act as suicide substrates. Such a covalent enzyme inactivation could possibly proceed by a Michael-type addition of a nucleophilic group in the enzyme to the α,β-unsaturated carbonyl.[17] However, a considerably larger enzyme preparation will be needed in order for the enzyme–inhibitor complex to be directly detected and its nature delineated.

In view of this binding behaviour, the dissociation constants (*K*_D_) were derived from simple titration experiments assuming that the enzyme–inhibitor complex is catalytically inactive.[18] For both compounds, within the experimental error, the *K*_D_ was found to be 0.94(±0.1)×10^−6^ m (Figure 2). It is interesting that peptides IΔA and IΔF are almost equally potent isomerase inhibitors, even though the saturated substrate peptides, IA and IF, differ strongly in their catalytic efficiencies. Given that the similar hydrophobicities of the *Z*/*E* isomers in IΔF account for similar active site binding, this fact suggests that the side chain substituent at the second position contributes only little to the transition state binding.

**Figure 2:**
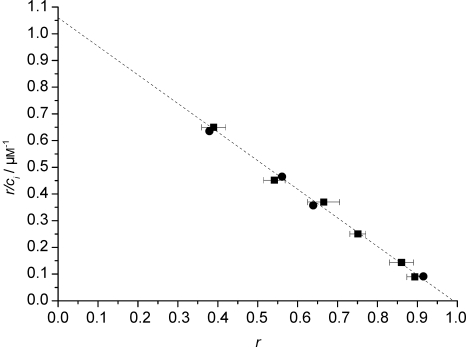
Scatchard plot for the determination of *K*_D_: *r* is the ratio of the enzyme activity with bound inhibitor IΔF (▪) or IΔA (•) to enzyme activity without inhibitor. Substrate was labelled peptide IF. Error bars represent standard deviations.

The proposed deprotonation/protonation mechanism necessarily implies the abstraction of a weak acidic proton by a weak basic catalytic site of the enzyme, such as the imidazole group of histidine, from the α-carbon of an amino acid residue in peptide linkage. The p*K*_a_ value of an α-proton has been estimated to be higher than 20 but might well approach the upper limit of enzyme-catalysed proton transfer reactions.[19] For the removal of such an α-proton, an enzyme could stabilize a planar enolate anion intermediate of the bound substrate (Scheme 2). Such a strategy has been demonstrated for the mandelate racemase, a member of the enolate superfamily, which engages the carbonyl group of mandelate in a low-barrier hydrogen bond and coordinates it to Mg^2+^.[20] Moreover, recent investigations on an isomerase from the venom of a spider, which catalyses the stereoinversion of a Ser within a peptide chain,[11] make an enolate anion a likely intermediate in this reaction[21] (Scheme 2).

Studies of the racemization of free amino acids have indicated that the reactant side chain group also contributes to the stability of the enolate anion. Aryl side chain groups, such as phenylglycine (Phg), can stabilize an intermediate enolate anion primarily by resonance.[22] In a similar manner, Met enhances the stability of the enolate by orbital overlap.[23] Indeed, studies on the enzyme-catalysed isomerization of various model peptides have shown that the highest observed relative reaction rates were obtained with peptides containing either of these residues at position two.[24]

A deprotonation/protonation mechanism is also utilized by several cofactor-independent amino acid racemases. Consistent with the idea of a planar transition state, these reactions are well known to be inhibited by planar intermediate analogues. An example is the inhibition of proline racemase by the planar compound pyrrole-2-carboxylic acid.[25] Furthermore, planar enamine and imine species have been shown to serve as potent inhibitors of the enzymes diaminopimelate epimerase and glutamate racemase.[26] Finally, the spider isomerase is also inhibited by a planar substrate analogue, which contains a Δ^2, 3^-Ala at the isomerization site.[21]

The platypus enzyme, on the other hand, was shown to be inhibited by amastatin but not by the related bestatin, both of which are competitive aminopeptidase inhibitors.[12, 27] The N-terminal residue of these compounds is either an aliphatic (amastatin) or an aromatic (bestatin) α-hydroxy-β-amino acid. Therefore, the different side chains were suspected to also determine the activity of the inhibitors and their binding to the active site.[27] Both compounds only have a similar, relatively weak effect (*K*_i_>10^−4^ m) on the reaction catalysed by the frog isomerase (Figure 3). It seems likely that those inhibitors with a β-amino acid bind to the active site in a substrate-like manner, albeit with the α^2^-carbon in an improper position for catalysis. Conversely, in the case of the unsaturated analogues, their tight binding to the enzyme as well as a high tolerance against the side chain of residue 2 suggest that they bind to the transition state conformation of the enzyme, once more emphasising the importance of geometry for catalysis.

**Figure 3:**
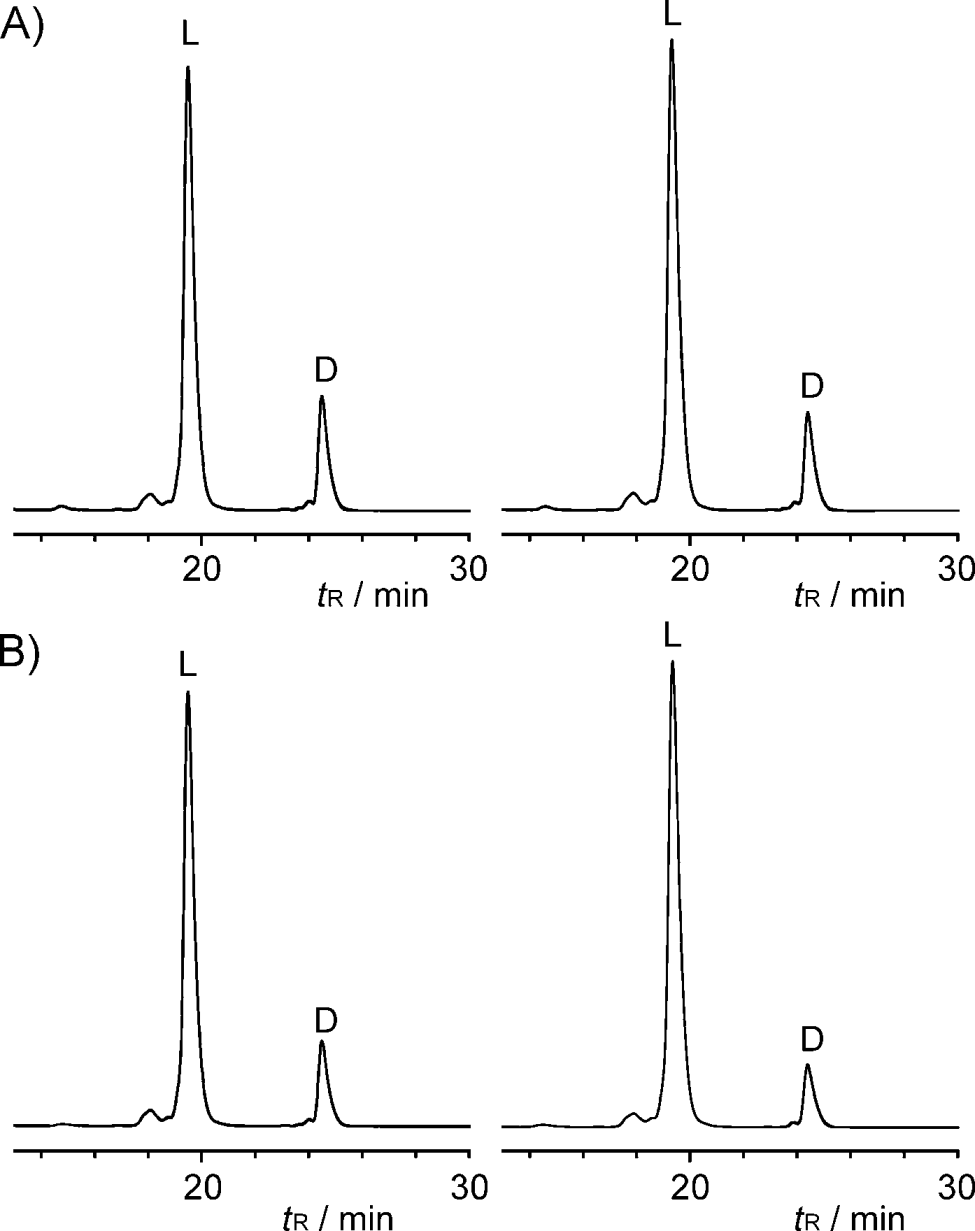
Inhibition by: A) bestatin, and B) amastatin. HPLC chromatogram after 10 min reaction of 100 μm labelled peptide IF (L) in the absence (left) or presence (right) of 100 μm inhibitor; product is represented by D.

Taken together, peptides containing an unsaturated amino acid as the second residue effectively inhibit the stereoinversion catalysed by the *Bombina* isomerase; this supports the notion of a planar transition state during this reaction. These are the first specific inhibitors to be described for this enzyme. These specific inhibitors supplement the set of tools available for studying this isomerase, which at present is very limited, mostly because attempts to express the enzyme in different over-expression systems have been unsuccessful. Therefore, these inhibitors could prove even more useful in future investigations of the post-translational isomerization of natural peptides.

## Experimental Section

**Synthesis of Ile-Δ^2, 3^-Ala-Gly-Pro-Val-Leu-amide (IΔA):** IΔA was synthesized with a selenocysteine (Sec)-containing peptide with subsequent oxidative elimination.[28] Thereby, Ile-Sec-Gly-Pro-Val-Leu-amide was synthesized by PSL (Heidelberg, Germany) on a continuous flow synthesizer using standard Fmoc solid phase chemistry with a Rink Amide AM resin (200–400 mesh, 0.62 meq g^−1^; Nova-Biochem), PyBOP (benzotriazol-1-yloxytripyrrolidinophosphonium hexafluorophosphate) as condensation reagent and *N*-methylmorpholine as a base. After cleavage, treatment with H_2_O_2_ (4 equiv in acetonitrile/H_2_O) yielded a dehydroalanine at the second position. The peptide was purified by reverse-phase HPLC over a C-18 column (Vydac) with a linear gradient of acetonitrile (solvent A, 0.1 % TFA; solvent B, 80 % acetonitrile) and their mass was determined by MALDI-MS. Found *m*/*z* 566.3 [*M*+H]^+^, calcd *m*/*z* 566.4 [*M*+H]^+^ (Figure S4 in the Supporting Information).

**Synthesis of Ile-Δ^2, 3^-Phe-Gly-Pro-Val-Leu-amide (IΔF):** The peptide IΔF was synthesized through modified Erlenmeyer–Plöchl and Bergmann syntheses.[29] Briefly, Boc-Ile-(dl-3-phenylserine) was synthesized by condensation of Boc-Ile-OH (1.0 g, 4.32 mmol) with dl-3-phenylserine (1.4 g, 5.52 mmol) by using *N*-methylmorpholine (477 μL, 4.33 mmol) and isobutyl chloroformate (566 μL, 4.33 mmol) in solution. The dipeptide (1.6 g, 4.07 mmol) was dried and ethylacetate extracted at pH 3. For the synthesis of the azlactone, freshly distilled acetic anhydride (3.34 mL) and anhydrous sodium acetate (0.33 g, 4.08 mmol) were added to the dipeptide. This yielded an azlactone, which was precipitated with crushed ice, recrystallised and dried. Azlactone (16.85 mg, 47.0 μmol) was coupled to solid-phase prepared Gly-Pro-Val-Leu-amide (2.26 mg, 5.9 μmol) in dichloromethane (1 mL) with triethylamine (50.2 μmol) and dried. The peptide was deprotected after purification by RP-HPLC with TFA (50 %) in acetonitrile and purified by RP-HPLC and resulted in 0.92 mg of IΔF (*Z* and *E* isomers could not be separated). The product was characterized by ESI-IT-MS (*m*/*z* 642 [*M*+H]^+^; Figure S4 in the Supporting Information) and NMR spectroscopy. The TOCSY spectrum is shown in Figure S5 in the Supporting Information. The chemical shifts of the resonances of the assignment are listed in Table S1 in the Supporting Information.

**Enzyme preparation:** Skin secretion from *Bombina orientalis* was collected and processed as described.[9] Briefly, the glycoprotein fraction eluted from ConA–Sepharose with methylmannoside was passed over a Sephacryl S-300 column (20 mm HEPES/NaOH buffer, pH 7.6, 1 mm EDTA), fractions with isomerase activity were subsequently passed over SP–Sepharose and the flow-through was fractionated over Q-Sepharose after adjustment to pH 8.6 with NaOH. The enzyme was eluted with a gradient of NaCl (1–2.5 mm in 20 mm HEPES/NaOH, 1 mm EDTA, pH 8.6). Active concentrated fractions were once more chromatographed over SP- and Q-Sepharose under the same conditions, and yielded considerably narrower peaks of activity. Every single fraction was then tested for enzymatic activity, and the protein's molecular weight was determined by SDS-PAGE. Highest enzymatic activity coincided with an apparently homogeneous protein of 52 kDa molecular mass. The enzyme concentration was 8.9 mg mL^−1^ as determined by UV absorption at 280 nm. The extinction coefficient *ɛ*=54 445 m^−1^ cm^−1^ was calculated with the ProtParam tool (http://expasy.org/tools/protparam.html) assuming that both Cys residues form cystines. The specific activity was 224 nmol h^−1^ mg^−1^ at 100 μm substrate IF, pH 6.5 and 37 °C.

**Deuterium incorporation experiment:** Substrate IF (100 μm) was incubated with enzyme (89 μg mL^−1^) in phosphate buffer (50 mm) in ^2^H_2_O containing EDTA (5 mm) at 37 °C at pD 6.5 in ^2^H_2_O. After 5, 10, 20, 30, 50, 70 and 90 min, aliquots of the solution were analysed by RP-HPLC (218TP C-18 column, Vydac, Hesperia, CA, USA) with a linear gradient of acetonitrile (solvent A, 0.1 % TFA; solvent B, 80 % acetonitrile), which was started after a delay of 5 min in order to remove bulk D_2_O. The UV absorbance was recorded at 214 nm. Substrate as well as product peaks were collected, vacuum-dried twice, and redissolved in order to remove deuterium incorporated into backbone amides. The isotope pattern was then quantitatively analysed on a MALDI-TOF/TOF 4800 Analyzer (AB Sciex, Canada) operated in reflector positive mode acquiring 1000–2000 total shots per spectrum with a laser intensity of 3000–3500 with α-cyano hydroxylcinnamic acid as a matrix. Spectra were acquired and processed by using 4800 Analyzer and Data Explorer Software.

**Inhibition experiments:** The substrate in the inhibition assays was either unlabelled peptide IF (Ile-Phe-Gly-Pro-Ser-Arg-Ser-amide) or Ile-Phe-Gly-Pro-Ser-Arg-Cys-amide (PSL), which was coupled via the SH group to the fluorophor Bodipy FL IA [(4,4-difluoro-5,7-dimethyl-4-bora-3′,4′-adiaza-*S*-indacene-3-propionyl)-*N*-iodoacetylethylenediamine; Invitrogen]. Substrate and inhibitors at the given concentrations were incubated with aliquots of the enzyme (44.5 μg mL^−1^) at 37 °C at pH 6.5 in phosphate buffer (50 mm) containing EDTA (5 mm) and analyzed by RP-HPLC (218TP C-18 column, Vydac, Hesperia, CA) with a linear gradient of acetonitrile (solvent A, 0.1 % TFA; solvent B, 80 % acetonitrile). Unlabelled peptides were detected by UV absorbance at 214 nm. Fluorescence emission of the labelled peptides was excited at 480 nm and recorded at 514 nm. Peaks were integrated with Beckman System Gold Software. Data from three independent experiments were combined. Some experiments were performed with peptide IA (Ile-Ala-Gly-Pro-Ser-Arg-Ser-amide) as a substrate.

Enzyme reactivation was tested by dilution of pretreated (50 μm inhibitor) enzyme into 100-fold volume buffer or 50 μm inhibitor (final enzyme concentration 22.25 μL mL^−1^) and subsequent determination of the isomerase activities with the standard assay. Additional controls were treated equally, except for the addition of inhibitor. For testing the effect of amastatin or bestatin (Sigma), the isomerization rate of labelled peptide IF (10 or 100 μm) was determined in the presence of inhibitor at concentrations equal to that of the substrate.
