# Supplementary material for: Tight Binding of Transition-State Analogues to a Peptidyl-Aminoacyl-l/d-Isomerase from Frog Skin
Source: Chembiochem. 2011 Jul 7;12(13):1996–2000. doi: 10.1002/cbic.201100203 (PMC3195878; doi:10.1002/cbic.201100203)
Supplement: Supplementary file 1 [file cbic0012-1996-SD1.pdf]

CHEMBIOCHEM

Supporting Information

© Copyright Wiley-VCH Verlag GmbH & Co. KGaA, 69451 Weinheim, 2011

Tight Binding of Transition-State Analogues to a Peptidyl-Aminoacyl-L/D-Isomerase from Frog Skin

Verena Gehmayr,[b] Christa Mollay,[c] Lorenz Reith,[b] Norbert Müller,[b] and Alexander Jilek*[a, b, c]

cbic_201100203_sm_miscellaneous_information.pdf

Supplemental material

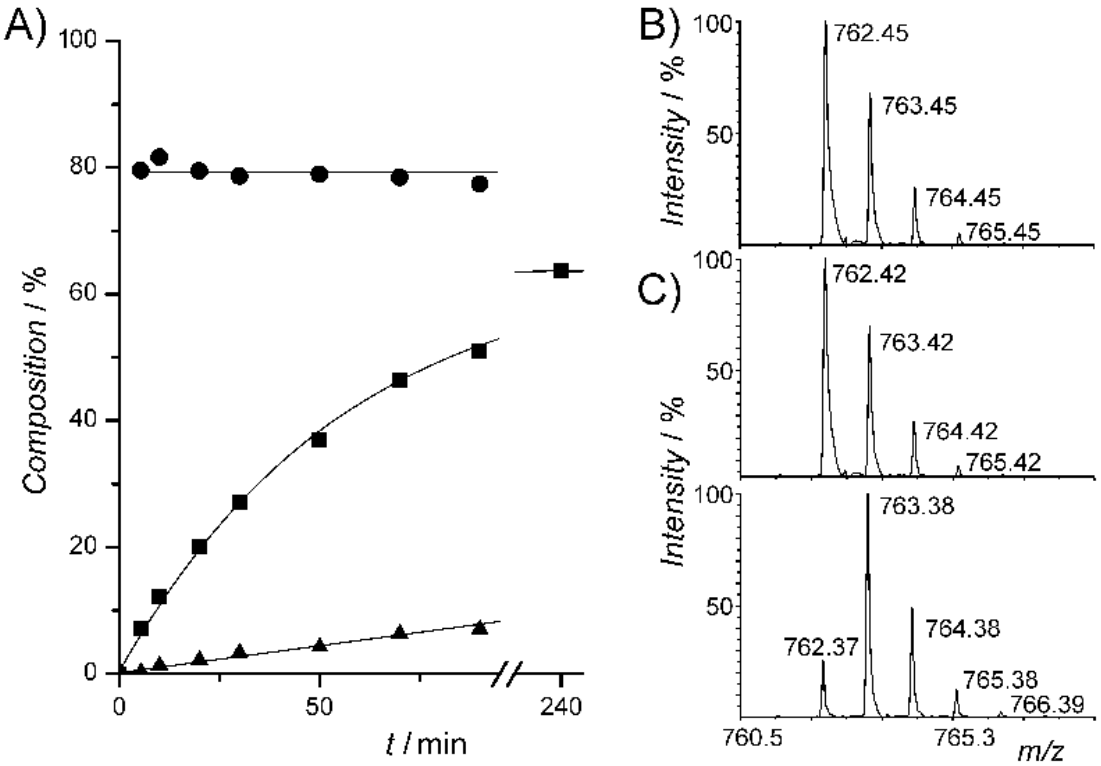

Fig. S1: Enzymatic reaction performed in deuterated water. A: Fraction of product (■) and fractions of deuterated substrate (▲) or product (●) during the isomerization of substrate IF (100 µM) at pD 6.5 at 37°C. Turnover at 240 min corresponds to an equilibrium constant K_{eq} =1.76 *versus* 2.85 in water. Residual water content was 6%. Fractions of deuterated peptide were calculated from mass peaks based on the observed isotope pattern (B) of IF. C: MALDI-MS of substrate (upper panel) and product (lower panel) fractions collected from HPLC after 10 min of reaction.

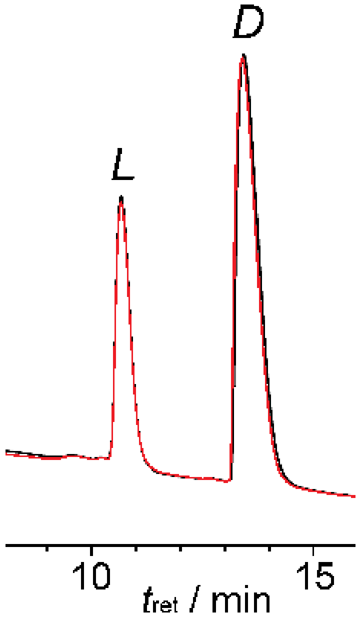

Fig. S2: Reaction equilibria in water (black graph)or in the presence of 1µM IΔA (red graph). Substrate was 500µM IF (*L*). Product is represented by *D*.

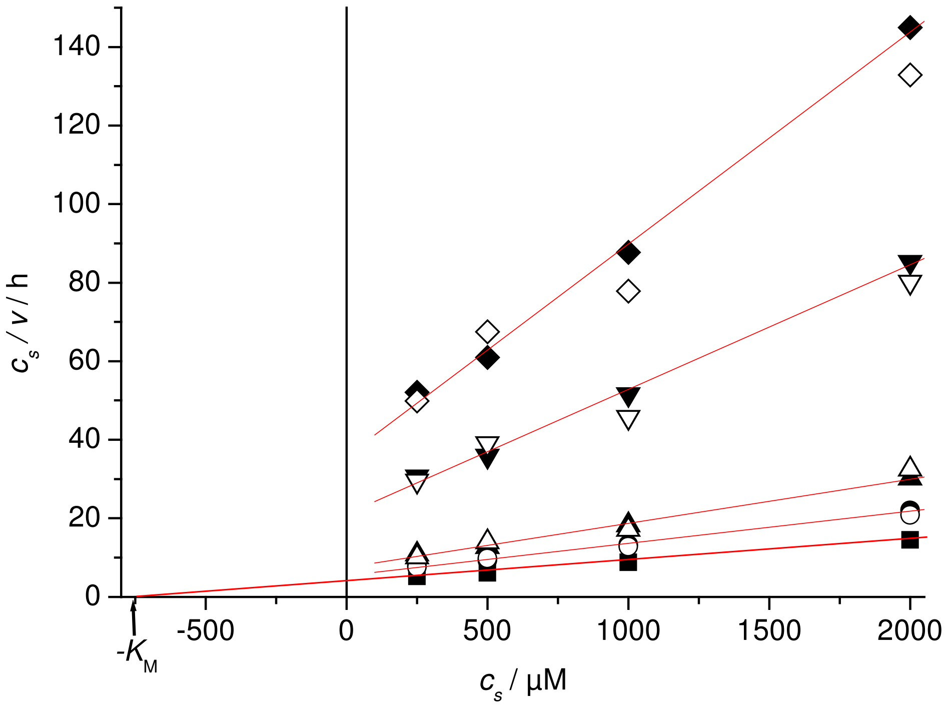

Fig. S3: Michaelis-Menten analysis of kinetic data. A Hanes-plot representation of initial reaction velocities as a function of substrate concentration is shown at various inhibitor concentrations. Without inhibitor (■) and in presence of 0.5 µM (●), 1 µM (▲), 5 µM (▼) and 10 µM (□) IΔF or 0.5 µM (○), 1 µM (□), 5 µM (□) and 10 µM (□) IΔA. Substrate was peptide IF.

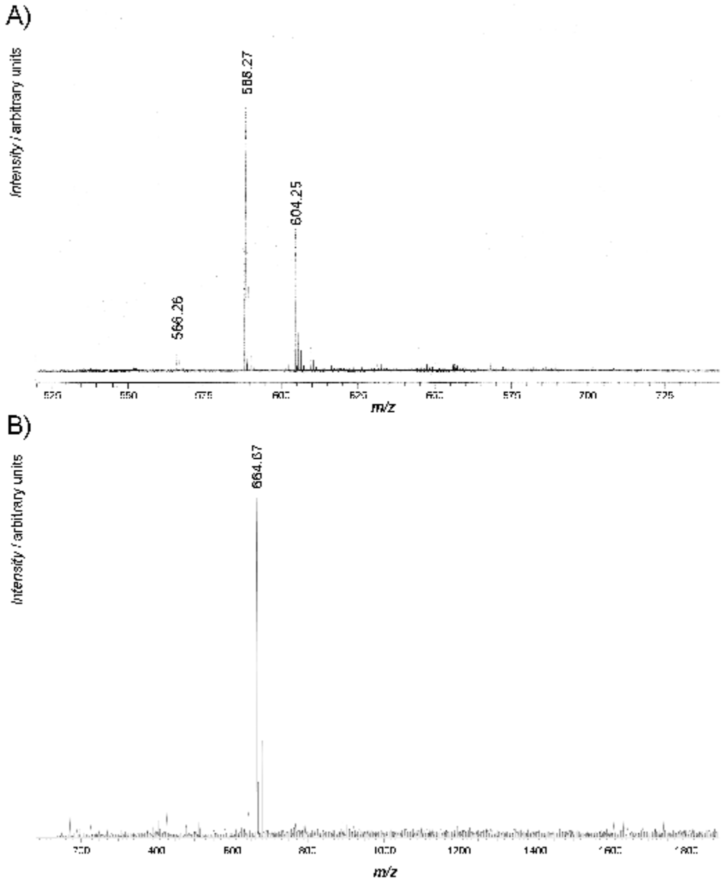

Fig. S4: Mass spectra of peptides IΔA (A) and IΔF (B). A: Observed m/z = 566.3 [MH+]. Other peaks are [MNa+] and [MK+]. B: Observed m/z = 664.7 [MNa+].

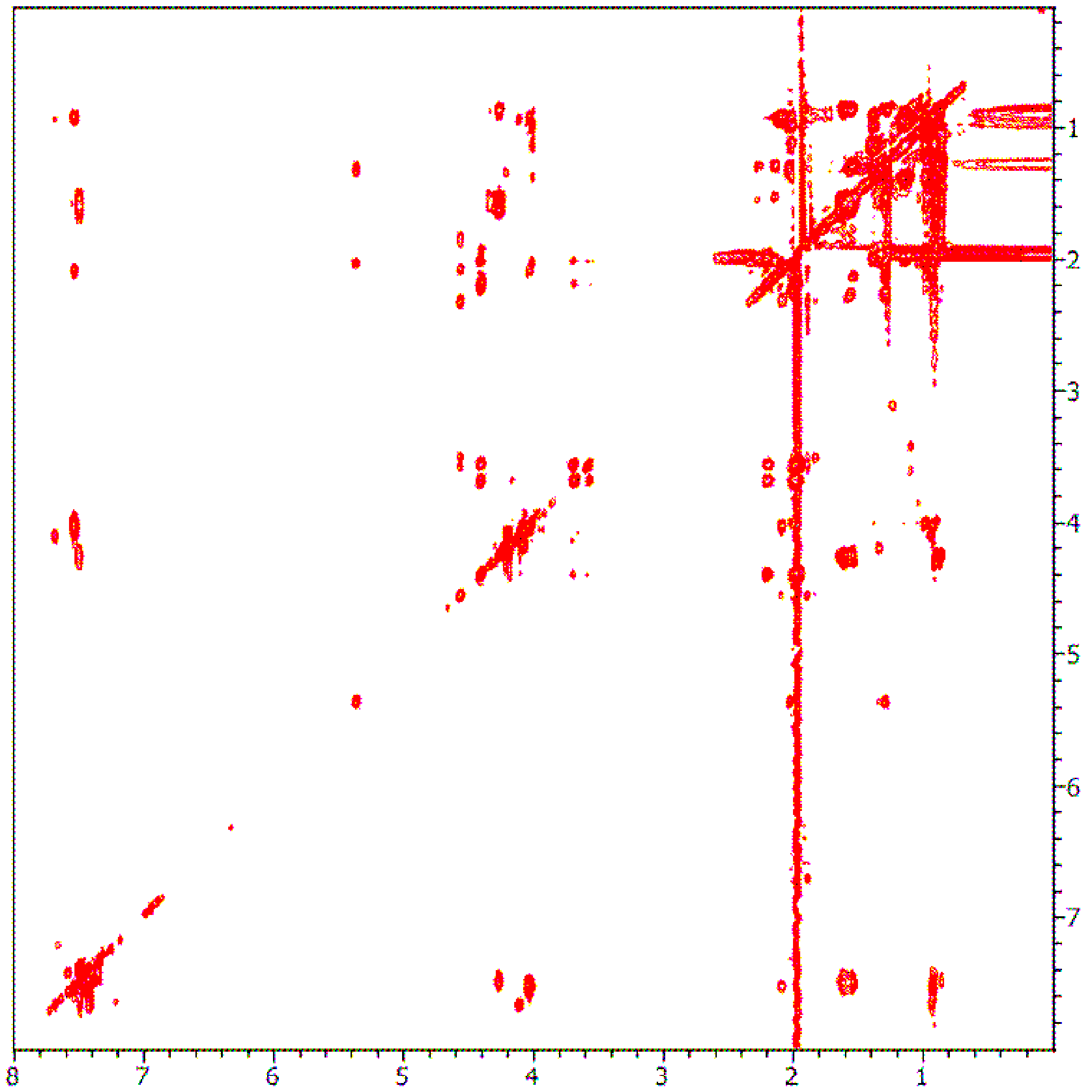

Fig. S5: TOCSY spectrum of peptide IΔF. NMR-spectra were recorded on a Bruker DRX 500 MHz instruments equipped with a cryogenically cooled probe (TXI). ^{1}H and TOCSY spectra (60ms mixing time) were used for resonance assignment. The acquired data set for the TOCSY spectrum consisted of 256 data points in t_1 and 4096 data points in t_2, recorded with 32 scans per increment. Each data matrix was zero filled to 512 x 8192 points and 82° shifted Cosine and 82° shifted Gaussian weighing functions were applied in f1 and f2 prior to Fourier transformation. All experiments were recorded at 300K in acetonitrile-d3, which was also used as reference for the chemical shift. The resonance assignment is listed in table S1.

Table S1: Resonance-signal assignment of peptide IΔF (**A**) from the spectrum in Fig.S1. **B**: An alternative set of resonance-signals indicates the presence of both *Z*- and *E*-isomers.

A)

residue	Amino Acid	Proton	ρ [ppm]
1	Ile	^{1}HA	4.01
		^{1}HB	2.00
		^{1}HD	0.98
		^{1}HG12	1.37
		^{1}HG13	1.13
		^{1}HG2	0.90
2	$\Delta^{2,3}$-Phe	^{1}HB	3.98
		^{1}HG	7.43
3	Gly	^{1}H-N	7.79
		^{1}HA2	4.19
		^{1}H A3	4.08
4	Pro	^{1}HA	4.56
		^{1}HB2	2.33
		^{1}HB3	2.07
		^{1}HD2	3.58
		^{1}HD3	3.51
		^{1}HG	1.87
5	Val	^{1}H-N	7.68
		^{1}HA	4.10
		^{1}HB	2.10
		^{1}HG1 + HG2	0.94
6	Leu	^{1}H-N	7.49
		^{1}HA	4.26
		^{1}HB2	1.65
		^{1}HB3	1.63
		^{1}HD1	0.91
		^{1}HD2	0.86
		^{1}HG	1.60

B)

4’	Pro	^{1}HA	4.40
		^{1}HB2	2.19
		^{1}HB3	1.99
		^{1}HD2	3.68
		^{1}HD3	3.56
		^{1}HG	1.99
5’	Val	^{1}H-N	7.53
		^{1}HA	4.02
		^{1}HB	2.08
		^{1}HG	0.92
